# A prediction nomogram for deep venous thrombosis risk in patients undergoing primary total hip and knee arthroplasty: a retrospective study

**DOI:** 10.1186/s12959-023-00538-8

**Published:** 2023-10-12

**Authors:** Zhencan Lin, Hao Sun, Deng Li, Zhiqing Cai, Zhencheng Huang, Fangzhou Liu, Meiyi Chen, Yimin Wang, Jie Xu, Ruofan Ma

**Affiliations:** 1 Department of Orthopedics, Sun Yat-sen Memorial Hospital, Sun Yat-sen University, Guangzhou, Guangdong China 510120 China; 2 Department of Orthopedics, The Eighth Affiliated Hospital, Sun Yat-Sen University, Shenzhen, Guangdong China 518000 China

**Keywords:** Total hip and knee arthroplasty (THA/TKA), Deep venous thrombosis (DVT), Thrombin antithrombin complex (TAT), Nomogram, Prognosis

## Abstract

**Introduction:**

Deep venous thrombosis (DVT) prediction after total hip and knee arthroplasty remains challenging. Early diagnosis and treatment of DVT are crucial. This research aimed to develop a nomogram for early DVT prediction.

**Methods:**

A total of 317 patients undergoing primary total hip and knee arthroplasty in Sun Yat-sen Memorial Hospital were enrolled between May 2020 and September 2022. Data from May 2020 to February 2022 were used as the development datasets to build the nomogram model (n = 238). Using multivariate logistic regression, independent variables and a nomogram for predicting the occurrence of DVT were identified. Datasets used to validate the model for internal validation ranged from March 2022 to September 2022 (n = 79). The nomogram’s capacity for prediction was also compared with the Caprini score.

**Results:**

For both the development and validation datasets, DVT was found in a total of 38 (15.97%) and 9 patients (11.39%) on post-operative day 7 (pod7), respectively. 59.6% patients were symptomatic DVT (leg swelling). The multivariate analysis revealed that surgical site (Knee vs. Hip), leg swelling and thrombin-antithrombin complex (TAT) were associated with DVT. The previously indicated variables were used to build the nomogram, and for the development and validation datasets, respectively. In development and validation datasets, the area under the receiver operating characteristic curve was 0.836 and 0.957, respectively. In both datasets, the predictive value of the Nomogram is greater than the Caprini score.

**Conclusions:**

A proposed nomogram incorporating surgical site (Knee vs. Hip), leg swelling, and thrombin antithrombin complex (TAT) may facilitate the identification of patients who are more prone to develop DVT on pod7.

**Supplementary Information:**

The online version contains supplementary material available at 10.1186/s12959-023-00538-8.

## Introduction

The incidence of joint disease is increasing rapidly as the global population ages in patients with osteoarthritis, rheumatoid arthritis, developmental dysplasia of the hip, and ankylosing spondylitis. To treat end-stage arthritis, total joint arthroplasty (TJA), which includes total hip arthroplasty (THA) and total knee arthroplasty (TKA), is a common and highly successful procedure. However, a common side effect following major orthopaedic surgery is venous thromboembolism (VTE), which includes pulmonary embolism (PE) and deep vein thrombosis (DVT) [1]. The prevalence of DVT in Europe and the United States is 2.22-3.29%, while the prevalence of PE is 0.87-1.99%, with fatal PE affecting 0.30% of patients [2, 3]. In Asia, the prevalence of DVT is 1.4% and PE is 1.1% [4]. Even with quick treatment, some patients develop post-thrombotic syndrome, characterized by chronic limb discomfort and swelling, which can severely impair their quality of life [5, 6]. Although various measures available to prevent thrombosis range from intermittent pneumatic compressive devices to various pharmaceutical agents: low molecular weight heparin, aspirin, warfarin, and factor Xa inhibitors, DVT events are still detected in patients after joint replacement. However, it should be noted that asymptomatic DVT is as high as 47%, and it may progress to life-threatening PE [7]. Based on this, it is important that early prevention and diagnosis of thrombosis.

At present, there are numerous thrombosis risk assessment scales, such as the Caprini score, Wells score. These scales have been validated and applied in clinical work. The Wells score has limitations in predicting the risk of PE in orthopaedic trauma patients and cannot identify patients who need CT pulmonary angiography (CTPA) to rule out PE [8]. The Caprini score has been validated in assessing the VTE risk of critically ill surgical patients, general surgery patients, and urologic surgery patients [9, 10]. For THA/TKA patients, some scholars consider Caprini score ≥ 10 as high-risk patients with Venous Thrombosis, which can be used for risk stratification [11, 12].

Among them, The Caprini score is the most prevalent surgical thrombus risk assessment model. However, recent studies have indicated that the Caprini score has limitations in evaluating VTE patients after joint replacement. This is primarily due to the inability of the Caprini score to provide a clear and quantifiable risk stratification, because all TJA patients are considered high-risk individuals (Caprini score ≥ 5). Therefore, we need to find better indicators or methods for early assessment of thrombosis risk. Nomogram is a visual statistical model built based on multivariate regression analysis, to describe how the variables in the prediction model are related [13, 14]. Its predictive value has been well-documented in tumor-related nomogram studies [15, 16]. Thus, our purpose was to build a predictive nomogram as a DVT risk model of primary TJA patients. Based on this nomogram, it could help identify patients at high risk for VTE and early interventions.

## Methods

### Study population

This study was approved by the ethics committee of Sun Yat-Sen Memorial Hospital, Sun Yat-Sen University, Guangzhou, China. The case data of 317 patients undergoing primary THA/TKA between May 2020 and September 2022 were collected and retrospectively analyzed at Sun Yat-sen Memorial Hospital. The inclusion criteria were as follows: patients ≥ 18 years of age; end-stage disease of the hip or knee; primary total joint replacement; complete case data. The exclusion criteria included the following: patients undergoing revision total joint; diagnosed VTE before surgery; antiplatelet or anticoagulant drugs were used before surgery. Data from May 2020 to February 2022 were used as the development datasets to build the model (n = 238). Datasets used to internally validate ranged from March 2022 to September 2022 (n = 79).

The indicators we collected were mainly Caprini score-related indicators, thrombin antithrombin complex (TAT, the first day after surgery, normal range: 0.00-0.55 mg/L FEU), and D-dimer (the first day after surgery, normal range: 0-4ng/mL). previous studies have shown that TAT level on pod1 may serve as predictor for DVT events on pod7 [17].

### Surgical procedure

All operations were performed by two senior orthopedic surgeons with at least 20 years of clinical experience. Tourniquets were consistently utilized in every TKA procedure. Patients were encouraged to engage in early mobility activities, such as walking on the ground, starting from the first day after the surgery on pod1 if their medical condition allowed. Intermittent pneumatic compressive devices (IPCD) were introduced to enhance blood flow return and prevent DVVT on pod2 before discharge, and stop using IPCD after DVT is diagnosed. Continuous passive motion (CPM) techniques were employed to promote joint flexibility and recovery following TKA. All patients received low molecular heparin (Enoxaparin Sodium) 0.4 mL (4000 AxaIU) subcutaneously once daily 12 h following surgery until a diagnosis of lower-extremity DVT was made or discharge.

### Laboratory evaluations and VTE monitoring

Blood samples were collected prior to surgery and on pod1. To diagnose or rule out DVT, all patients had a Doppler ultrasonography of the lower-extremity prior to surgery and again on pod7. The primary study outcome was diagnosed lower-extremity DVT on pod7. In this study, deep venous thrombosis and pulmonary embolism were considered VTE. The specific deep veins that were analyzed included the external iliac vein, the femoral vein, the popliteal vein, the posterior tibial vein, the anterior tibial vein, the peroneal vein, and the muscular calf vein. All of these veins are located in the lower extremities.

### Statistical analysis

**Fig. 1:**
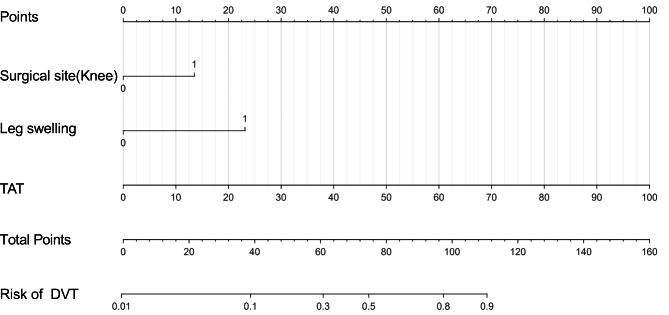
Nomogram for predicting the probability of DVT

To assess the VTE risk after primary THA/TKA, various statistical analyses were conducted. The study compared two continuous variables using the two-sample t-test and analyzed 17 categorical variables using the chi-square test. Subsequently, univariate binary logistic regression was performed to analyze the variables that showed a p-value < 0.05 in the previous analyses (Table 1). These significant indicators were then incorporated into the multivariate logistic regression in development datasets. The multivariate logistic regression is shown in Table 2. Figure 1 displays a nomogram based on development datasets and the results of multivariate logistic regression (p < 0.05) that visualizes the risk of thrombosis. To assess the nomogram’s ability to predict DVT, the receiver operating characteristic curve (ROC), a calibration plot (1000 bootstrap resamples), and decision curve analysis (DCA) were performed for internal validation. Pairwise comparisons of AUC-ROC were performed to compare nomograms and Caprini scores in each dataset. Furthermore, the categorical net reclassification improvement (NRI) and the integrated discrimination improvement (IDI) were utilized to measure the extent to which the nomogram’s predictive power exceeded that of the Caprini score. The clinical usefulness of the nomogram and Caprini score was evaluated using decision curve analysis (DCA), which measures the net gain of different threshold probabilities in both the development and validation datasets.

**Table 1:** Univariate logstic regression of development dataset.

Variable	OR	OR 95% CI	p-value
**Surgical site(Knee)**	3.354	1.511–7.446	0.003*
**Gender(Female)**	0.443	0.164–1.195	0.108
**Age**			
**41–60**	0.661	0.12–3.652	0.635
**61–74**	1.847	0.393–8.694	0.437
**≥ 75**	0.591	0.088–3.954	0.588
**BMI ≥ 25**	2.756	1.244–6.106	0.013*
**Malignancy**	2.057	0.52–8.136	0.304
**Varicose vein**	-	-	0.999
**Positive blood test**	-	-	0.999
**History of DVT**	-	-	0.999
**Smoking**	0.685	0.15–3.127	0.625
**Diabetes need insulin**	-	-	0.999
**HRT**	0.208	0.027–1.589	0.130
**Frature of the Hip**	1.266	0.401–3.996	0.687
**Bed for 72h**	1.572	0.656–3.767	0.310
**Transfusion**	0.791	0.370–1.690	0.545
**CVC**	0.874	0.102–7.472	0.902
**Leg swelling**	4.021	1.961–8.246	< 0.0001*
**COPD**	5.378	0.329–87.91	0.238
**TAT**	1.065	1.041–1.089	< 0.0001*
**D-dimer**	1.041	1.016	< 0.001*

*p < 0.05

**Table 2:** Multivariable logstic regression of develoment dataset.

Variable	β-Coefficient	SE	OR	OR 95% CI	p-value
**Intercept**	-6.257	NA	NA	NA	NA
**Surgical site(Knee)**	1.215	0.538	3.37	1.174–9.678	0.024*
**BMI ≥ 25**	0.2	0.519	1.221	0.442–3.374	0.7
**Leg swelling**	1.627	0.462	5.089	2.057–12.59	< 0.001*
**TAT**	0.063	0.013	1.065	1.038–1.093	< 0.001*
**D-dimer**	-0.015	0.018	0.985	0.951–1.019	0.379

*p < 0.05

All analyses were performed using statistical software R version 4.2 and the result was considered statistically significant at the p < 0.05 level of significance. The “rms” program package was used to construct the nomogram and calibration plot; calculate NRI and IDI using the package “Predicate ABEL”; and use the “rmda” package to establish the DCA.

## Results

### General characteristics

**Fig. 2:**
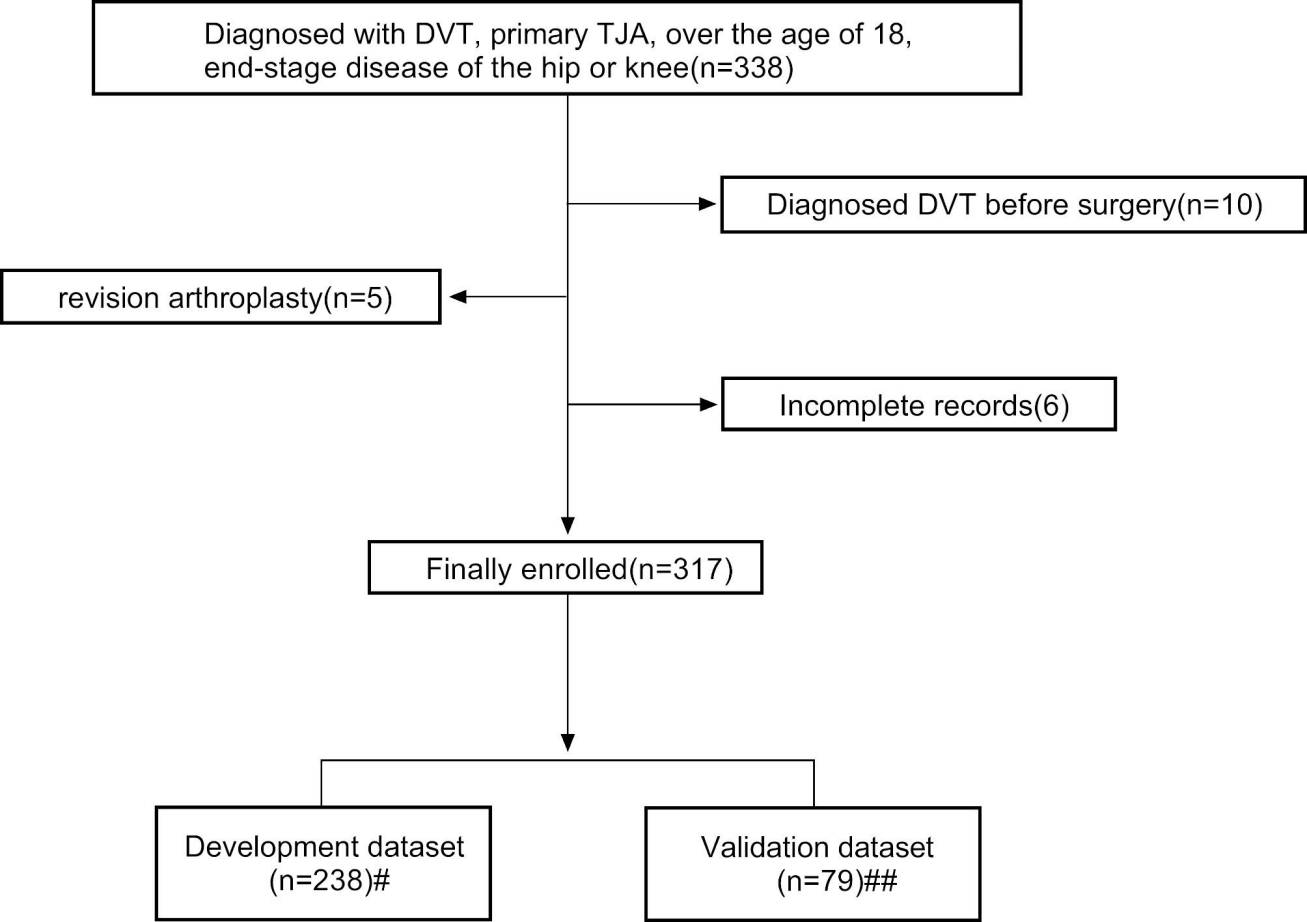
Case screening and datasets partitioning flow diagram #Data from May 2020 to February 2022 were used as the development datasets to build the model(n = 238). ##Datasets used to validate the model for internal validation ranged from March 2022 to September 2022(n = 79)

The inclusion of patients is depicted in Fig. 2.A total of 317 patients who met the eligibility criteria were included in the study. Lower-extremity DVT occurred in 14.83% (47/317) of the enrolled patients and 59.6% were symptomatic DVT (leg swelling). No patients were diagnosed with PE or had PE-related symptoms. 238 of the 317 patients were included in the development datasets, while the remaining 79 were included in the validation datasets, following the rules depicted in Fig. 2. Patients were divided into two groups (non-DVT group and DVT group) based on the development of DVT.

Several factors were collected to assess their relationship with DVT development. These factors included age, sex, body mass index (BMI), surgical site, history of malignancy, varicose vein, positive blood test, history of DVT, smoking, diabetes need insulin, hormone replacement therapy (HRT), fracture of the hip, bed for 72 h, blood transfusion, central venous catheter (CVC), leg swelling, chronic obstructive pulmonary disease (COPD), Protopathy, thrombin antithrombin complex (TAT), D-dimer. These baseline characteristics of the patients are summarized in Table 3.

**Table 3:** Baseline characteristics of non-DVT group vs. DVT group.

	Entire dataset			Development dataset (n=238)			Validation dataset		
	non-DVT n = 270	DVT n = 47	p-value	non-DVT n = 200	DVT n = 38	p-value	non-DVT n = 70	DVT n = 9	p-value
**Surgical site(Knee)**	131(48.5%)	37(78.7%)	< 0.0001	98(49.0%)	29(76.3%)	0.002*	33(47.1%)	8(88.9%)	0.045
**Gender(Female)**	204(75.6%)	40(85.1%)	0.151	149(74.5%)	33(86.8%)	1	55(78.6%)	7(77.8%)	1
**Age**									
**41–60**	82	6	0.029*	59	6	0.187	23	0	0.72
**61–74**	122	35	0.001*	95	27	0.026*	27	8	0.017*
**≥ 75**	49	4	0.201	33	3	0.362	16	1	0.507
**BMI ≥ 25**	118(43.7%)	30(63.8%)	0.01*	81(40.5%)	24(63.2%)	0.032*	37(52.8%)	6(66.7%)	0.703
**Malignancy**	12(4.4%)	3(6.4%)	0.837	8(4.0%)	3(7.8%)	0.531	4(5.7%)	0	1
**Varicose vein**	4(1.5%)	0	1	2(1.0%)	0	1	2(2.9%)	0	1
**Positive blood test**	10(3.7%)	0	0.368	7(3.5%)	0	0.518	3(4.3%)	0	1
**History of DVT**	2(0.7%)	0	1	2(1.0%)	0	1	0	0	-
**Smoking**	21(7.8%)	3(6.4%)	0.972	15(7.5%)	2(5.3%)	0.883	6(8.6%)	1(11.1%)	0.586
**Diabetes need insulin**	4(1.5%)	0	1	4(2.0%)	0	1	0	0	-
**HRT**	32(11.9%)	1(2.1%)	0.04*	23(11.5%)	1(2.6%)	0.171	9(12.9%)	0	0.558
**Frature of the Hip**	28(10.4%)	5(10.6%)	0.956	17(8.5%)	4(10.5)	0.927	11(15.7%)	1(11.1%)	1
**Bed for 72h**	43(15.9)	10(21.3%)	0.364	29(14.5%)	8(21.1%)	0.307	14(20.0%)	2(22.2%)	1
**Blood Transfusion**	98(36.3%)	15(31.9%)	0.563	68(34.0%)	11(28.9%)	0.544	30(42.6%)	4(44.4%)	1
**CVC**	8(3.0%)	1(2.1%)	1	6(3.0%)	1(2.6%)	1	2(2.9%)	0	1
**Leg swelling**	64(23.7%)	28(59.6%)	< 0.0001*	47(23.5%)	21(55.3%)	< 0.0001*	17(24.3%)	7(77.8%)	< 0.0001*
**COPD**	1(0.4%)	2(4.3%)	0.059	1(0.5%)	1(2.6%)	0.294	0	1(11.1%)	0.114
**Protopathy**	129/9/51/31/30/20	31/4/2/3/5/2	0.036*	99/7/38/25/19/12	24/3/2/3/4/2	0.232	30/2/13/6/11/8	7/1/0/0/1/0	0.214
**TAT**	21.6 ± 13.8	47.4 ± 27.6	< 0.0001*	21.8 ± 13.3	41.4 ± 22.0	< 0.0001*	20.9 ± 15.1	72.5 ± 35.4	< 0.0001*
**D-dimer**	10.8 ± 10.6	20.5 ± 18.2	< 0.0001*	10.9 ± 10.8	18.9 ± 17.4	< 0.0001*	10.7 ± 9.8	28.3 ± 21.0	< 0.0001*

Protopathy: OA/RA/AVN/DDH/FNF/Others. OA, osteoarthritis; RA, rheumatic arthritis; AVN, avascular necrosis; DDH, developmental dysplasia of the hip; FNF, fracture of neck of femur

*p < 0.05

As shown in Table 3, eight variables exhibited significant differences between the non-DVT group and the DVT group. These variables included the surgical site (Knee vs. Hip) (p < 0.001), age (p < 0.05), BMI ≥ 25 (p < 0.05), HRT (p < 0.05), Leg swelling (p < 0.001), TAT (p < 0.001), and D-dimer (p < 0.001). None of the following variables resulted in a significant difference between the two groups: malignancy, varicose vein, positive blood test, history of DVT, smoking, diabetes need insulin, fracture of the hip, bed for 72 h, blood transfusion, CVC, COPD.

### Screening for predictive factors

We performed univariate logistic regression for each variable in the development datasets to identify possible DVT risk factors (Table 1). The initial multivariable logistic regression contained those five variables with p < 0.05. In the end, the logistic regression model included these three of them: surgical site (Knee vs. Hip), leg swelling, and TAT. In the final model, all terms related to DVT development were statistically significant (p < 0.05, Table 2).

### Risk prediction nomogram development

Using the results of multiple logistic regression, these three characteristics were incorporated into a model, and a nomogram was developed to calculate the probability of lower-extremity DVT (R²= 0.35, C-index = 0.836, Fig. 1). The predictor of TAT has the greatest impact, followed by the presence of leg swelling, and the surgical site (Knee vs. Hip) had the least effect on lower-extremity DVT development in this nomogram.

### The nomogram’s predictive power and net benefit versus the caprini score

**Fig. 3:**
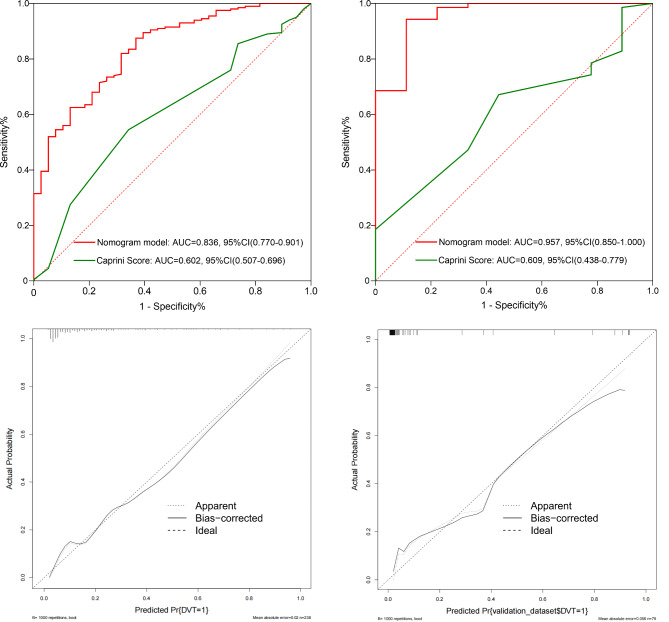
Decision curve analysis of our nomogram and Caprini score in the development(A) and validation dataset (B)

The DCA showed superior performance compared to the predictive model in terms of net benefit (Fig. 3A). In the nomogram’s internal validation, it also has a large value of AUC. Additionally, the DCA curve demonstrated a significant net benefit of the nomogram model in both the validation datasets and overall (Fig. 3B).

**Fig. 4:**
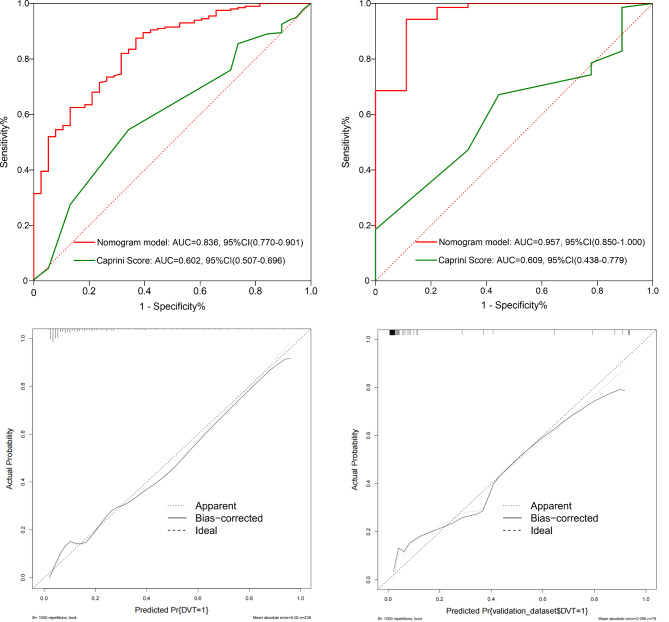
The discriminative power and precision of the nomogram is detected by ROC curves and calibration plot (**A**)(**C**) development datasets, (**B**) (**D**) validation datasets

To comprehensively compare the classic Caprini score with the nomogram, we compared the AUC of the two models. The results showed that the prediction ability of nomogram had better than that of Caprini score (0.836 vs. 0.602, p<0.001; Fig. 4A). Similar results were also observed in the validation datasets (0.957 vs. 0.609, p<0.001; Fig. 4B). The nomogram showed improved prediction performance over Caprini, on both NRI and IDI (development datasets: categorical NRI: 0.499(0.285–0.713), p < 0.0001; IDI: 0.259 (0.165–0.352), p < 0.0001; validation datasets: categorical NRI: 0.739 (0.459–1.011), p < 0.0001; IDI: 0.619 (0.376–0.861), p < 0.0001; Table 4).

**Table 4:** Comparison of the nomogram model and the Caprini Score.

	Development dataset			Validation dataset		
	Caprini Score	Nomogram	p-Value	Caprini Score	Nomogram	p-Value
**AUC(95%CI)**	0.602(0.507–0.696)	0.836(0.770–0.901)	< 0.0001*	0.602(0.438–0.779)	0.957(0.850-1.000)	< 0.0001*
**Categorical**						
**NRI(95%CI)**	Reference	0.499(0.285–0.713)	< 0.0001*	Reference	0.739(0.459–1.011)	< 0.0001*
**IDI(95%CI)**	Reference	0.259(0.165–0.352)	< 0.0001*	Reference	0.619(0.376–0.861)	< 0.0001*

Abbreviations: AUC, area under curve; CI, confidence interval; NRI, net reclassification improvement; IDI, integrated discrimination improvement. *p < 0.05

## Discussion

Deep vein thrombosis of the lower-extremity is a common complication of hip and knee arthroplasty. DVT imposes a huge burden on both individuals and society [18]. Consequently, it is crucial to focus on predicting and preventing the occurrence of DVT in order to improve patient outcomes. In this study, we analyzed the clinical data of patients undergoing primary THA/TKA from our centers in the past two years. Our goal was develop a clinical prediction model that could assess the risk of lower-extremity DVT in these patients. In clinical practice, the predictors used in this model are common and readily identifiable, including clinical symptoms and laboratory indicators. In both the development and validation datasets, our final model exhibited predictive ability (c-index = 0.836). This model will provide evidence for the prediction of lower-extremity DVT event in patients undergoing primary THA/TKA in a clinical setting.

Similar to previous research, the incidence of DVT was 14.83% on pod7 in this study [19]. Previous studies have shown that advanced age and obesity are important risk factors for thrombosis, which seems to be inconsistent with our results. That may be due to the sample size or the fact that age and obesity were recorded as categorical variables rather than continuous variables according to the Caprini score.

In recent years, some new coagulation related indicators have emerged, such as thrombin-antithrombin complex (TAT). Antithrombin regulates blood clotting by binding to thrombin [20]. Elevated TAT indicates overproduction of thrombin and can indicate a high risk of thrombosis. The half-life of thrombin is too short to be measured directly, so TAT can be measured indirectly to react with thrombin level to reflect the activation of coagulation [21]. Some studies have pointed out that TAT can be used as a indictor of hypercoagulability [20]. Our results further supported the reference value of TAT level in the early diagnosis of lower-extremity DVT. In our nomogram model, the TAT level made the main contribution, and the risk of thrombosis gradually increased with the increase of the TAT level. When the value of TAT is greater than 60ng/mL, the incidence of thrombosis will exceed 30% without considering other risk factors.

In our study, TKA had a higher risk of lower-extremity DVT than THA, which is in line with earlier research showing that TKA has a twice-as-high risk of DVT as THA [22]. We believe that the reasons for this are as follows. First, TKA has longer operation times usually and the use of tourniquets compared with THA [23]. Second, greater pain and increased painkiller use after TKA may be reduced mobility and increased thrombosis [24].

We also found that lower limb swelling after surgery was also a risk factor for lower-extremity DVT. However, making a definitive clinical diagnosis of DVT is challenging due to the lack of consistently reliable clinical signs or symptoms. Pitting edema, loss of the bony prominences, obscured surface foot veins, or indentation of the leg are common symptoms of these patients [25]. In this study, 59.6% patients were symptomatic DVT (leg swelling), but 23.7% with leg swelling without DVT (Table 3). Several studies have shown that lower limb swelling is associated with DVT because emboli can affect venous return and cause swelling [26]. Decreased postoperative activity and increased bed rest also affected venous return [27].

Previously, D-dimer levels have been identified as a sensitive predictor of venous thromboembolism in patients [28, 29]. Although D-dimer was statistically significant between the DVT and non-DVT groups, it was not ultimately included in our prediction model. This was due to the large standard deviation of D-dimer levels, causing significant fluctuations in its values, both in DVT and non-DVT patients. As its predictive power is diminished. THA/TKA must lead to marked fibrinolysis and a significant increase in D-dimer, a fibrin degradation product [30].

It is worth noting that certain indicators, such as sex, history of malignancy, varicose veins, positive blood tests, history of DVT, smoking, diabetes requiring insulin, hormone replacement therapy, hip fracture, 72-hour bed rest, blood transfusion, central venous catheter (CVC), and chronic obstructive pulmonary disease (COPD), were excluded from our study. The lower incidence of these events among our patients made further statistical analysis difficult.

Nevertheless, our study has several limitations. First, this is a single-center, retrospective study with inherent limitations intrinsic to the retrospective nature of the study. Second, low molecular weight heparin (Enoxaparin Sodium) 0.4 mL (4000 AxaIU) was administered to all patients until a diagnosis of lower-extremity DVT was made. However, there are still many anticoagulant drugs or regimens available, such as factor Xa inhibitors, vitamin K antagonists or warfarin. Hence, in future studies, larger sample size and a multicenter design were needed to illustrate these findings. At the same time, we also need to verify the role of the model under different anticoagulation options. Finally, the patients are all Asian. Our model must be validated in populations of other ethnicities.

## Conclusion

In summary, our study revealed that several factors, including surgical site (Knee vs. Hip), leg swelling and TAT, were found to be significant predictors of lower-extremity DVT on pod7 in patients undergoing THA/TKA. Building upon these findings, we developed a nomogram that incorporated these factors to provide a predictive tool for assessing the risk of lower-extremity DVT. We found that the performance of our nomogram in this study was satisfactory for DVT risk assessment. At the same time, the newly derived nomogram appeared to be superior to the traditional Caprini score model in predicting lower-extremity DVT. While the present findings are hypothesis generating requiring adequate validation, they suggest the proposed nomogram might aid therapeutic decisions and clinical monitoring in patients.

### Electronic supplementary material

Below is the link to the electronic supplementary material.

Supplementary Material 1

## Data Availability

All other data can be obtained from the authors upon reasonable request.
