# Supplementary material for: A prediction nomogram for deep venous thrombosis risk in patients undergoing primary total hip and knee arthroplasty: a retrospective study
Source: Thromb J. 2023 Oct 12;21:106. doi: 10.1186/s12959-023-00538-8 (PMC10568791; doi:10.1186/s12959-023-00538-8)
Supplement: Supplementary file 1 — Supplementary Material 1 [file 12959_2023_538_MOESM1_ESM.docx]

Positive blood test include factors: Factor V Leiden/activated protein C resistance, antithrombin III deficiency, protein C & S deficiency, dysfibrinogenemia, 20210A prothrombin mutation and acquired factors: lupus anticoagulant, antiphospholipid antibodies, myeloproliferative disorders (including thrombocytosis), disorders of plasminogen and plasmin activation, heparin-induced thrombocytopenia, hyperviscosity syndromes, and homocysteinemia, HIV infection.

Leg swelling: measure the circumference of the lower limbs (10 cm above the patella and 10 cm below the patella) before and every day after the surgery. If the circumference increases ≥ 3cm before the operation, it is considered as leg swelling.

laboratory measures of D-dimer and TAT: Blood samples were collected from venous blood, mixed with 0.129 mol/L sodium citrate (9:1 ratio), and centrifuged to obtain plasma for determination. The detection of D-dimer was carried out by immunoturbidimetric method by the Japan Sysmex series hemagglutination instrument (model: Sysmex-CA7000). The detection of TAT is carried out with an automatic hemagglutination instrument (model: Sysmex HISL-5000) and supporting reagents.
